# Effects of a participatory community quality improvement strategy on improving household and provider health care behaviors and practices: a propensity score analysis

**DOI:** 10.1186/s12884-018-1977-9

**Published:** 2018-09-24

**Authors:** Tewabech Wereta, Wuleta Betemariam, Ali Mehryar Karim, Nebreed Fesseha Zemichael, Selamawit Dagnew, Abera Wanboru, Antoinette Bhattacharya

**Affiliations:** 1 The Last Ten Kilometers Project (L10K) 2020, JSI Research and Training Institute, Inc, Bole Sub-City, Kebele 03/05, Hs # 2111, Addis Ababa, Ethiopia; 2 Department of Disease Control, Faculty of Infectious and Tropical Disease, London School of Hygiene and Tropical Medicine, Keppel Street, London, WC1E 7HT UK

**Keywords:** Maternal, Newborn, Quality improvement, Community engagement

## Abstract

**Background:**

Maternal and newborn health care intervention coverage has increased in many low-income countries over the last decade, yet poor quality of care remains a challenge, limiting health gains. The World Health Organization envisions community engagement as a critical component of health care delivery systems to ensure quality services, responsive to community needs. Aligned with this, a Participatory Community Quality Improvement (PCQI) strategy was introduced in Ethiopia, in 14 of 91 rural *woredas* (districts) where the Last Ten Kilometers Project (L10 K) Platform activities were supporting national Basic Emergency Obstetric and Newborn Care (BEmONC) strengthening strategies. This paper examines the effects of the PCQI strategy in improving maternal and newborn care behaviors, and providers’ and households’ practices.

**Methods:**

PCQI engages communities in identifying barriers to access and quality of services, and developing, implementing and monitoring solutions. Thirty-four intervention *kebeles* (communities), which included the L10 K Platform, BEmONC, and PCQI, and 82 comparison kebeles, which included the L10 K Platform and BEmONC, were visited in December 2010–January 2011 and again 48 months later. Twelve women with children aged 0 to 11 months were interviewed in each kebele. Propensity score matching was used to estimate the program’s average treatment effects (ATEs) on women’s care seeking behavior, providers’ service provision behavior and households’ newborn care practices.

**Results:**

The ATEs of PCQI were statistically significant (*p* < 0.05) for two care seeking behaviors — four or more antenatal care (ANC) visits and institutional deliveries at 14% (95% CI: 6, 21) and 11% (95% CI: 4, 17), respectively — and one service provision behavior — complete ANC at 17% (95% CI: 11, 24). We found no evidence of an effect on remaining outcomes relating to household newborn care practices, and postnatal care performed by the provider.

**Conclusions:**

National BEmONC strengthening and government initiatives to improve access and quality of maternal and newborn health services, together with L10 K Platform activities, appeared to work better for some care practices where communities were engaged in the PCQI strategy. Additional research with more robust measure of impact and cost-effectiveness analysis would be useful to establish effectiveness for a wider set of outcomes.

**Electronic supplementary material:**

The online version of this article (10.1186/s12884-018-1977-9) contains supplementary material, which is available to authorized users.

## Background

Maternal and neonatal mortality have reduced considerably in the last decades, but low and middle-income countries continue to bear the highest burden of deaths among women around the time of childbirth and among newborns [1]. Of the 830 women who died each day in 2015 due to complications related to pregnancy and childbirth, the majority were in Sub-Saharan Africa and Southern Asia [2]. Although maternal deaths declined by 44% between 1990 and 2015, the maternal mortality ratio in many countries remains unacceptably high with disparities among and within countries [1].

Of the estimated 5.9 million child deaths in 2015, 40% occurred during the neonatal period. The decline in neonatal mortality in 1990–2015 has been slower than that of post-neonatal under-five mortality (1–59 months) in most low and middle-income countries [3].

Sustainable Development Goal 3 aims to reduce the global maternal mortality ratio to less than 70 per 100,000 live births by 2030 and the newborn mortality rate to as low as 12 per 1,000 live births [4]. Achieving these ambitious targets will require universal coverage of high quality and high impact maternal and newborn health interventions [5, 6].

Evidence from the Millennium Development Goal era indicates that although many countries were able to increase the coverage of a number of high impact interventions (e.g. skilled birth attendance), poor quality of care remains a concern limiting health gains, as it reduces access to care and its effectiveness [6–10]. Thus, implementing high quality interventions during the intrapartum and immediate postpartum period —to prevent the majority of the maternal and newborn deaths [5]— is among the most urgent priorities of the global action agendas [11, 12].

Among the strategies essential for achieving improvements in maternal and newborn health, as reiterated by various international declarations and statements, is the engagement of households and communities in health interventions. Working with individuals, families and communities is a critical link to ensure the recommended continuum of care throughout pregnancy, childbirth and the postpartum period. Given the growing recognition that health cannot be assured solely by actors in the health sector, engaging communities is also key to improving and maintaining interventions that advance health gains [13–16].

People are more likely to use and respond positively to health services if they have been involved in decisions about how these services are delivered. They are willing to contribute resources for health improvement and they would also be more likely to change behaviors that would help them take control over their own lives. Furthermore, community participation in setting health priorities, making decisions, and planning and implementing strategies helps to promote health and quality of service [13, 17, 18].

Community engagement refers to efforts that promote dialogue, sharing of information and resources, and decision making between members of the community and the health department [19]. It improves mutual understanding and increases awareness of the realities, perspectives and conditions of the other party. It enables communities to effectively identify problems and root causes, understand context, plan and manage resources, solve problems, and use data to monitor progress and make decisions. This participatory approach helps to raise awareness within the community and to stimulate social support and participation in problem-solving [13, 17, 18, 20].

Several studies report that engaging communities improves care seeking behavior, including increased antenatal care (ANC) visits and institutional deliveries [20–22]. A systematic review of the effects of community participation on improved maternal and newborn health that included 12 countries with multiple interventions studies in different parts of the same country was conducted by Marston et al. [23]. Studies in Nepal, India, Uganda, Kenya and Eritrea reported that engaging communities had a largely positive impact in terms of increased use of ANC services, facility deliveries, reduced neonatal mortality and improved accountability of health care providers. Moreover, engaging communities in health programs can lead to improvements in client confidence, trust-building, credibility and, subsequently, to improved perceptions of health care quality. This may result in increased health care seeking behavior and uptake and quality of services, ultimately improving health outcomes [19, 24]. Community engagement can also create accountability and promote a sense of ownership, acceptability of health policies by community members and sustainability of quality improvement interventions [23, 24].

Nearly 40 years after the Declaration of Alma-Ata, which stated equity, social justice and community participation as key to achieving primary health care, the application of community engagement in improving the delivery of health care remains sporadic and poorly documented [15].

While the role of community engagement in improving maternal and newborn health services has been demonstrated, the variation of community engagement approaches and the intensity of implementation make it difficult to determine which of the strategies were most effective and compare the different strategies [25–27]. Further, because of these variations in community engagement approaches and because community engagement is usually implemented within a strategy with multiple components, some studies have been unable to directly identify the link between community participation and improved health outcomes [26]. Some have argued that the relationship between community engagement and health outcomes is not direct as there are other factors that positively influence the relationship, and others note that the success community engagement depends on the context and cannot be replicated on a larger scale [19, 27].

The objective of this study was to assess the contribution of a Participatory Community Quality Improvement (PCQI) strategy on improving household and provider maternal and newborn care behaviors and practices in Ethiopia. This paper is the third in a series of four papers investigating community-based strategies to improve reproductive, maternal, newborn, and child health in the country. The other three papers studied the Women’s Development Army (WDA), Community-Based Data for Decision-Making (CBDDM) and the Family Conversation strategies [28].

## Methods

### Study setting

Ethiopia’s maternal mortality ratio and newborn mortality rate are among the highest in the world at 421 per 100,000 live births and 29 per 1,000 live births, respectively, according to the 2016 Ethiopia Demographic and Health Survey estimate [29]. Although access to maternal and newborn health services has improved, the quality of care remains an immense challenge and often the services do not respond fully to community needs [30, 31].

In 2015, the Government of Ethiopia made a commitment towards achieving the health-related Sustainable Development Goals by launching its Health Sector Transformation Plan, under which it set ambitious targets to reduce the maternal mortality ratio to 199 per 100,000 live births and the neonatal mortality rate to 10 per 1,000 live births by 2020 [7]. Moreover, the Health Sector Transformation Plan cited universal coverage of high quality maternal and newborn health services which respond to the community’s needs and are respectful to clients, among its top priorities.

The Government of Ethiopia launched a number of programs to increase access to quality maternal and child health care. For example, it introduced its flagship Health Extension Program in 2004 to improve primary health care at community level and transfer ownership of and responsibility for improving health to communities and individual households through a package of promotive, preventive and basic curative services aimed at women and children [31].

The primary level of healthcare, as articulated in the Health Sector Transformation Plan, is the primary health care unit which comprises four or five health posts and one health center, which together with three or four other primary health care units is served by a primary hospital. The primary health care unit is appointed to serve as the administrative, technical and supportive supervision link to their health posts [7]. Each health post is staffed by two Health Extension Workers (HEWs) and, to extend their reach in mobilizing communities and households, each *kebele* (community) includes a network of women volunteers who form the WDA, also known as Health Development Army.

Since 2008, The Last Ten Kilometers Project (L10 K) has been working to improve coverage of effective reproductive, maternal, newborn and child health services, and to strengthen the skills of HEWs in 115 of 408 *woredas* (districts) across four regions of Ethiopia, covering about 19% of the country’s population: Amhara, Oromia, Tigray, and Southern Nations, Nationalities, and Peoples’ Region. The L10 K Platform in the 115 woredas included CBDDM, a surveillance system of households to ensure continuum of care for reproductive, maternal, newborn and child health services; Family Conversations, a forum conducted at the house of a pregnant woman with her family members during the antenatal period, to reinforce birth preparedness; and Birth Notification to ensure early postnatal care. (For details on the Platform please see Additional files 1–3 for the first paper in this supplement and two of the other papers in this supplement on the CBDDM and Family Conversations strategies [28, 32, 33]).

In October 2012, the L10 K Project introduced a program in 91 woredas to improve basic emergency obstetric and newborn care (BEmONC) through training, mentoring, provision of equipment and supplies, and addressing barriers for improved infection prevention practices. The L10 K Project complemented Ethiopian government initiatives to improve maternal and newborn health outcomes, which included infrastructure expansion of primary health care units, strengthening the referral system, procurement of ambulances to provide free transport to laboring women, maternal death surveillance and response, training of health care providers in basic maternal and neonatal care, and fee waivers and exemptions for maternal and child health care services [34].

To improve demand and quality of these services, the PCQI strategy was implemented from October 2012 in all 93 intervention *kebeles* communities) across 14 woredas where BEmONC was also initiated. The primary focus of PCQI in the first 18 months was on maternal care in two PCQI cycles (cycles described below); this was subsequently extended to include newborn care in the third cycle, for 6 months.

**Fig. 1:**
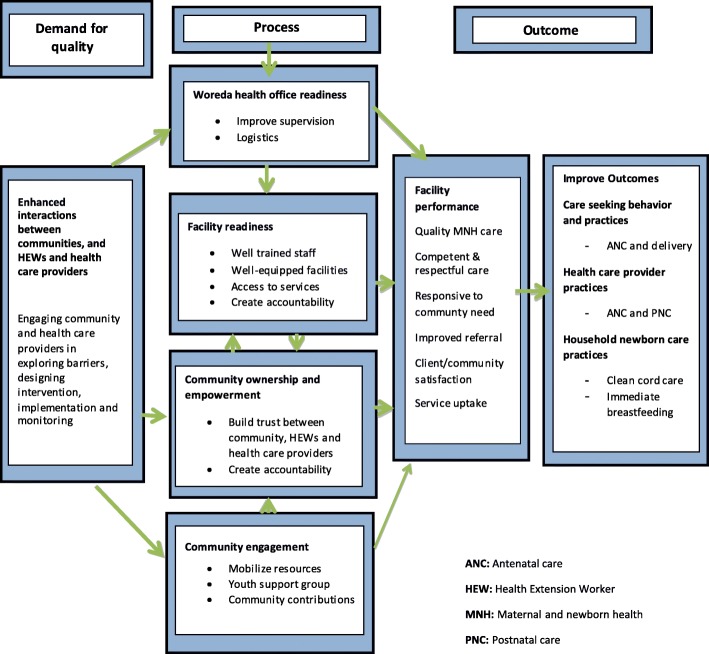
Conceptual framework of the PCQI strategy

The PCQI strategy aimed to achieve its goal by facilitating community involvement in defining, implementing and monitoring the quality improvement process. Figure 1 shows a conceptual framework for the strategy. Increasing access and quality of ANC, delivery and perinatal outcomes began with an understanding of the barriers to quality care from provider and community perspectives. By strengthening communication processes on issues related to the quality of maternal and neonatal care, it was expected that enhanced interactions between the communities, HEWs, health care providers and woreda health offices would lead to recognition of a shared responsibility in improving maternal and newborn care behaviors and practices.

**Fig. 2:**
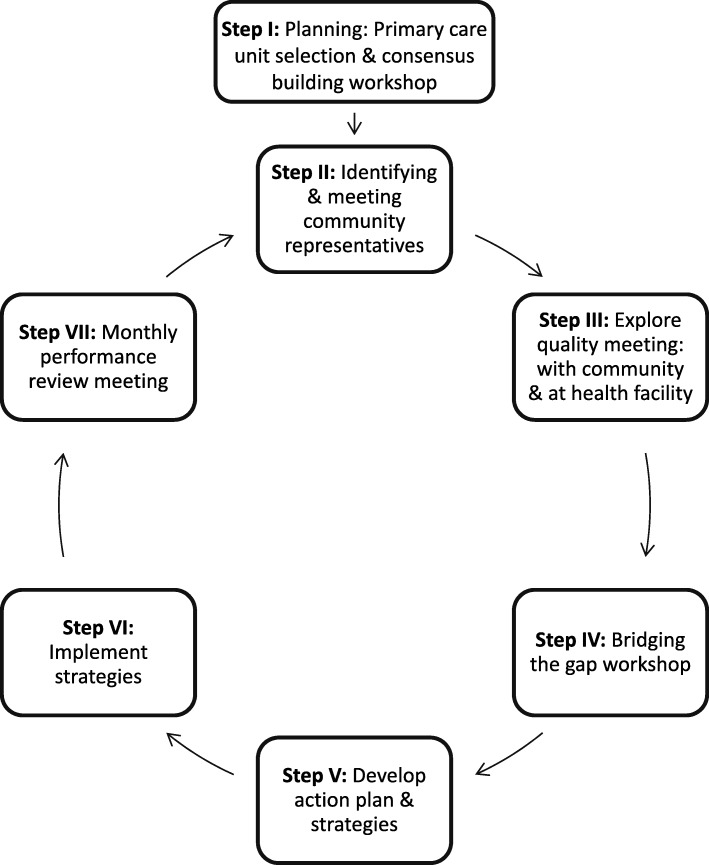
Participatory Community Quality Improvement cycle

PCQI involves a seven step cyclical process to achieve outcomes, as shown in Fig. 2.; 1) Selecting the primary care unit and holding a launching workshop with key stakeholders to build consensus. 2) Identifying and meeting community representatives (pregnant women, husbands, mother in laws; religious leaders and WDA members). 3) Explore quality meeting: conducting meetings with community representatives and biannual meetings at the facility level to identify major barriers to accessing services and gaps in service provision. 4) Bridging the gap workshops bringing community representatives and health workers from the health center together, to present their own perspective on barriers and service gaps (e.g. low care seeking behaviour; a health post not providing 24 h services because the HEWs live outside the kebele; communities unable to reach the health center due to poor roads and lack of transport; disruption of drugs and basic supplies; little confidence in giving birth at a health facility; and low levels of trust in the community). 5) Development of strategies and a joint action plan to address these barriers and gaps. 6) Implementation of the identified strategies (e.g. building HEW residences to enable HEWs to provide 24 h services; outreach sites; maintaining roads to facilitate transportation of pregnant mothers; preparing local stretchers for transporting women in labor and organizing youth groups to carry women in labor; supporting HEWs to inform the community about their schedule regularly; arranging a labor ward tour for women in their third trimester; training WDA members on the proper use of the family health card; promoting timely provision of drugs and supplies by woreda health offices). 7) Monthly review meetings of the performance of each strategy.

### Study design

This study was nested in a broader program evaluation for the L10 K Project and drew from the before-and-after household surveys conducted in January 2010–February 2011 and January 2014–February 2015, comparing areas with both PCQI and BEmONC strengthening in addition to the L10 K Platform, to the areas with BEmONC strengthening with the L10 K Platform alone. As indicated earlier, the L10 K Platform included the CBDDM and Family Conversation strategies and Birth Notification. The endline household survey was conducted after 26 months of PCQI intervention activities.

### Sample size and study participants

The sample size for the PCQI intervention area was based on precision and not based on detecting effect estimates of the PCQI strategy. The parameters of the sample size estimation were: 50% expected prevalence, 95% confidence interval (CI) with ±6 percentage-points precision, 1.5 cluster survey design effect, with number of respondents per cluster set at 12. Thus, 34 primary sampling units were needed to obtain the sample size for the intervention area. The study participants were women of reproductive age who had a live birth in the 12 months before the survey.

About 324 kebeles from the L10 K intervention areas were visited during both the survey periods of the broader evaluation (Table A2 in Additional file 1 for the first paper in this supplement [28]). These included 34 required kebeles from the PCQI areas and 82 kebeles that met the comparison group criteria. Thus, the sample sizes for propensity score matching (PSM) were 408 women from the intervention area and 990 women from the comparison area. In few cases the interviewers mistakenly interviewed more than 12 women from a kebele which resulted in six more women than the expected 984 (=12 X 82) respondents in the comparison areas.

### Data collection

The broader L10 K evaluation was a two-stage cluster survey, stratified by administrative regions and the L10 K Project strategy (including PCQI). Kebeles were selected as primary sampling units (clusters) with the probability proportionate to its population size. At the second stage, the sampling strategy described by Lemeshow and Robinson was used to select the household with the target respondents [35]. Accordingly, the first household was selected randomly from the middle of the kebele and then every fifth household was visited, moving away from the middle, and if the household had women with children aged 0 to 11 months old they were interviewed, after seeking their consent. Twelve women were interviewed from each kebele to obtain information on their socio-demographic background and the maternal and newborn health care behavior and practices associated with their most recent pregnancy and childbirth. The health post of the sampled kebeles was visited, the HEWs interviewed, and the health post records reviewed to obtain information on HEW to population ratio (Additional files 1–3 for the first paper in this supplement [28]).

### Outcomes of interest

The outcome indicators of interest were household and provider maternal and newborn health care behaviors and practices associated with the most recent childbirths among women with children aged 0 to 11 months. These were measured by the household survey. The definitions of the indicators are shown in Table 1.

**Table 1:** Definition of maternal and newborn health care indicators

Indicator	Definition
Women’s care seeking behavior	
Received four or more antenatal care visits	The percentage of women who went to a health facility for antenatal care at least four times during last pregnancy
Delivery at health facility	The percentage of women who had their last childbirth at a health facility with skilled birth attendants
Providers’ service provision behavior	
Complete antenatal care	The percentage of women who had their blood pressure measured, blood tested and urine tested during last pregnancy
Early postnatal care	The percentage of women who were visited by HEWs at home for postnatal care or newborn care within 48 h of last childbirth (among facility and home births. Women discharged from health facilities 6 hours after delivery)
Women’s care seeking and providers’ service provision behavior	
Neonatal tetanus protected childbirth	The percentage of women whose last childbirth was protected against neonatal tetanus
Households’ newborn care practices	
Practiced clean cord care of their newborn	The percentage who were not assisted by skilled birth attendants, but who cut the umbilical cord of their last newborn with a sterile instrument, tied the cut end of the cord with sterile thread and applied nothing to the cut end of the umbilical cord
Immediate initiation of breastfeeding	The percentage of women who were not assisted by skilled birth attendants, but who initiated breastfeeding their newborn immediately after birth

### Independent variables

The independent variables of interest were the indicator variables for each study arm and survey period and the respondent’s age, education, marital status, parity, religion, household wealth, distance of the respondent’s household to the nearest health facility, administrative region and HEW density of the sampled kebele.

The wealth index score was constructed for each household using the principal component analysis of household possessions (electricity, watch, radio, television, mobile phone, telephone, refrigerator, table, chair, bed, electric stove and kerosene lamp), and household characteristics (type of latrine and water source). The index was created among all respondents in the larger dataset from which the data for this study were extracted. The households of the larger survey were ranked according to the wealth score and then divided into five quintiles [36]. The WDA density in a kebele was the ratio between the total number of households and the number of active WDA team leaders in that kebele. Active WDA team leaders were those who had met with a HEW and discussed Health Extension Program issues during the 3 months preceding the survey.

### Statistical analysis

First, we compared individual, household and kebele-level sample characteristics measured in the follow-up survey across study arms using Pearson’s chi-squared statistics adjusted for cluster survey design effects. Similar statistical tests were done to 1) compare the outcome variables between the study arms during the baseline and the follow-up surveys; and 2) to assess statistically significant changes in the outcome variables during the observation period within each of the study arms. Stata 14.2 was used for the statistical analysis conducted for this study [37].

Propensity score matching was used to identify comparison individuals that were similar to those in the intervention area at follow-up, in terms of the background characteristics of the respondents, including kebele level estimates of the outcome of interest at baseline. Propensity scores are the probabilities of participation in the intervention and were estimated using logit models for each of the seven outcomes of interest, using the baseline kebele-level estimate of the given outcome as a covariate along with the background characteristics of the respondents in each case. We assessed average treatment effects (ATEs) of PCQI on the outcomes of interest using Stata’s ‘*teffects psmatch*’ procedure [38, 39]. The method imputes missing potential outcomes for each participant by using an average of the outcomes of similar participants that receive the other treatment level. The ATE was then calculated by taking the average of the difference between the observed and potential outcomes for each participant.

To assess the adequacy of the matching, we assessed the balance of covariates across study arms after matching. Balance was considered adequate if the standardized differences of the covariates between the study arms were less than 10% after matching [40]. A minimum of one-to-one match per participant was considered adequate if the balancing property was satisfied. If the one-to-one match did not satisfy the balancing property, then the minimum number of matches per participant was incrementally increased until the balancing property was satisfied [40]. The method selected an extra match per participant if the propensity score was tied. ATEs are presented from propensity scored matched models that satisfied the balancing property.

## Results

Table 2 indicates that compared with the comparison areas, the respondents from the intervention areas were more likely (*p* < 0.05) to have higher education, be from higher wealth quintiles, and live closer to a health facility.

**Table 2:** Characteristics of the complete sample by study arm during the follow-up survey (2014–15)

Sample characteristics		Comparison		Intervention		p-value
		%	N	%	N	
Age group	15–19	8	74	9	38	0.230
	20–24	26	254	31	126	
	25–34	51	501	46	186	
	35–49	16	160	15	59	
Education	Cannot read	59	584	47	193	0.009
	Primary	23	224	24	100	
	Higher	18	182	28	116	
Marital status	Other	2	17	3	11	0.247
	In union	98	973	97	397	
Number of children	1	26	253	31	124	0.104
	2	15	150	19	79	
	3	16	155	15	60	
	4+	44	431	35	144	
Religion	Orthodox	52	519	57	233	0.835
	Protestant	27	270	23	93	
	Muslim	19	192	20	80	
	Other	1	9	1	2	
Wealth quintile	Lowest	18	182	17	71	0.020
	Second	19	192	18	71	
	Middle	23	223	12	50	
	Fourth	23	228	24	96	
	Highest	17	166	29	120	
Distance to any health facility	< 30 min	46	457	60	243	0.003
	30 to 59 min	37	361	32	129	
	1+ hours	17	172	9	36	
Region	Tigray	18	175	17	71	0.953
	Amhara	25	250	29	117	
	Oromia	24	241	20	81	
	SNNP	33	324	34	140	
HEW density (population per HEW in kebele)	2,499	41	408	26	107	0.120
	2,500 to 3,499	22	215	42	171	
	3,500 to 4,999	25	251	21	86	
	5,000+	12	115	11	44	
Total		100	990	100	408	

*HEW* health extension worker, *SNNP* Southern Nations, Nationalities and Peoples

In terms of outcomes, coverage levels were generally higher in intervention groups than in comparison groups at baseline, with differences being statistically significant for complete ANC and institutional deliveries (*p* < 0.05, Table 3). In Table 3, comparing the baseline survey estimates with the follow-up survey estimates for the outcomes of interest, we can see that there were substantial improvements (*p* < 0.05) in all outcomes except early postnatal care (PNC) and clean cord care in both the study arms (*p*-values are not shown in Table 3). The clean cord care actually significantly declined (*p* < .05) between the survey periods in both the study arms.

**Table 3:** Maternal and newborn care by study arm and survey period, complete sample

	Baseline			Follow-up		
	Comparison % (N) (95% CI)	Intervention % (N) (95% CI)	*p*-value (α)	Comparison % (N) (95% CI)	Intervention % (N) (95% CI)	*p*-value (α)
ANC 4+	30 (983)	35 (408)	0.360	51 (990)	61 (408)	0.014
	(26, 25)	(26, 43)		(46, 55)	(54, 68)	
Complete ANC	10 (983)	18 (408)	0.007	52 (990)	74 (408)	< 0.001
	(7, 13)	(12, 23)		(46, 58)	(67, 81)	
Neonatal tetanus protected birth	56 (983)	61 (408)	0.293	65 (990)	72 (408)	0.063
	(52, 61)	(54, 68)		(60, 69)	(66, 79)	
Delivery at health facility	9 (983)	20 (408)	0.001	53 (990)	69 (408)	0.007
	(7, 12)	(13, 26)		(47, 60)	(60, 78)	
Early PNC	8 (983)	11 (408)	0.225	9 (990)	7 (408)	0.437
	(6, 11)	(7, 15)		(6, 11)	(4, 10)	
Clean cord care	47 (878)	46 (334)	0.862	35 (436)	28 (131)	0.223
	(41, 53)	(38, 54)		(28, 42)	(18, 37)	
Immediately initiating breastfeeding	60 (878)	60 (334)	0.941	71 (436)	71 (131)	0.934
	(54, 65)	(52, 69)		(64, 77)	(57, 86)	

(α) p-values of the test of differences between study arms

*ANC* antenatal care, *ANC 4+* received four or more antenatal care visits, *PNC* postnatal care

The ATEs of PCQI on household and provider maternal and newborn health care behaviors and practices from the PSM models are provided in Table 4. The PSM models were balanced for differences in the co-variates between the study arms (shown in Table 2) and the differences of the outcome between the two study arms at baseline (shown in Table 3). The standardized differences in the co-variates between intervention and comparison group respondents before and after matching for the seven PSM models are given in an additional table (Additional file 1 for this paper). The ATEs of PCQI on maternal and newborn behavior and practices were found to be significantly higher for three of the monitored outcomes in intervention sites compared with comparison sites; 14% and 11% points higher for women’s care seeking behavior, for four or more ANC visits and for institutional deliveries respectively, and 17% higher for health care providers provision of complete ANC (*p* < 0.05) (Table 4). There was no effect on the other outcomes.

**Table 4:** Maternal and newborn health care outcomes and average treatment effects, matched sample

	Control		Intervention		# of matched (α)	ATE		
	%	(N)^a^	%	(N)^a^		%-points	95% CI	*p*-value
ANC 4+	51.3	(372)	65.2	(941)	1–2	13.9	(6.3, 21.4)	< 0.001
Complete ANC	53.6	(372)	70.7	(941)	2–4	17.1	(10.7, 23.6)	< 0.001
Neonatal tetanus protected birth	69.9	(373)	69.6	(941)	1–3	−0.3	(−7.3, 6.7)	0.935
Institutional deliveries	57.6	(373)	68.1	(941)	4–5	10.5	(4.2, 16.8)	0.001
Early PNC	9.2	(373)	8.5	(941)	2–3	−0.7	(−4.9, 3.6)	0.753
Clean cord care	35.8	(119)	32.1	(415)	3–4	−3.6	(−14.7, 7.4)	0.521
Immediate initiation of breastfeeding	73.5	(119)	76.4	(534)	3–4	2.8	(−4.7, 10.6)	0.468

^a^Number of observation that was used for the PSM analysis

(α) Number of matches per participant for the PSM models

*ANC* antenatal care, *ANC 4+* received four or more antenatal visits, *ATE* average treatment effect, *PNC* postnatal care, *PSM* propensity score matching

## Discussion

This study found that community engagement in quality improvement for maternal and newborn health care services alongside the L10 K Platform activities and national BEmONC strengthening initiatives was associated with an increase in the coverage of ANC visits, complete ANC and institutional deliveries. Furthermore, there were several government initiatives such as skills training for health care providers, introduction of free ambulance services and maternal death surveillance targeted to improve access and quality of maternal health services in both the study arms and it appears that these initiatives work better where communities engage in the PCQI strategy.

These findings support the hypothesis that engagement of communities in quality improvement could promote women’s health care seeking and improve health care providers’ behavior in relation to quality of care. This is consistent with a study in Ethiopia which found that enhanced interactions among health workers and women and their families improved coverage and quality of maternal and newborn health services and outcomes [22].

It is also in line with the findings of a systematic review of effects of Community Participation on improving uptake of skilled care for maternal and newborn health which revealed community engagement increased uptake of ANC and institutional delivery in India, Nepal, Bangladesh, Uganda and Kenya [23]. Moreover, studies have showed that improvements in provider training, management practice, availability of equipment and supplies, communication with the community about the quality of care and cultural practices in relation to pregnancy and child birth increase care seeking for ANC, institutional delivery and the quality of those services [41, 42].

Along with L10 K-supported BEmONC activities and CBDDM, there were several government initiatives, such as staff training and maternal death surveillance, targeted to improve maternal services in both the study arms and it appears that these initiatives work better where communities engage in the PCQI strategy.

While the importance of addressing the needs of the supply side is undeniable, our study showed that the engagement of communities can play a significant role in enhancing health care seeking behaviors and practices and improving quality of care. Communities’ engagement in planning, implementation and decision-making can enable them, together with health system staff, to address barriers to care seeking, demand high quality health and make health systems responsive to communities, ultimately contributing to improved maternal and newborn health.

During a qualitative process evaluation of the L10 K project, communities also described the benefits of community engagement in quality improvement as community empowerment or ownership, more respectful care and an improved relationship between HEWs and their community (McCutcheon JC, Gebrekirstos T. A community quality improvement approach to facilitate more respectful care for pregnant women and increase health worker-assisted deliveries in rural Ethiopia. [unpublished]).

There was no evidence of an intervention effect on household practices of neonatal care and provision of early postnatal care by health care providers. This might be explained by the fact that the PCQI strategy focused on maternal services during the first 18 months of implementation and only expanded its focus to neonatal care practices and postpartum care 8 months before the evaluation. The short duration of exposure did not allow the PCQI cycles for newborn care to be completed. Moreover, the maternal and newborn outcomes considered were associated with the most recent childbirths among women with children aged 0 to 11 months; as such, one-third of respondents during the follow-up survey were not exposed to the PCQI cycles on newborn care.

It should also be noted that household newborn health care practices are deep-rooted cultural habits, having been practiced for generations and with great meaning attached to them, such as washing a newborn immediately after delivery because it is believed babies are born dirty and have to be cleaned. Communities therefore, are usually not willing to let go of these cultural practices, thus, necessitating gradual changes in practicing essential newborn health care. This may also be true of maternal care practices; however, this study did not address these practices [43–45].

With regards to study methods, we observed that a number of the background characteristics, including the outcomes of interest, were significantly different between the intervention group and comparison group respondents. As such, straightforward comparisons of changes in the outcomes of interest between the two study groups, such as difference-in-difference analysis, did not appear appropriate to estimate program effects. PSM has gained popularity to estimate intervention effects when participants differ between intervention and comparison groups [40]. We thus applied PSM models for estimating intervention effects. The intervention area individuals who did not have individuals with a similar covariate pattern in the comparison area were excluded from the analysis. By contrast, the difference-in-difference analysis does not exclude such individuals. The best possible analysis would have been combining difference-in-difference analysis with PSM models. However, our data did not permit it.

The major limitation of this study is that the ATEs estimated from PSM models do not account for unmeasured confounders and selection bias. For example, if the Government of Ethiopia’s ambulance program was significantly better in the intervention areas compared with the comparison areas, then the effects of the intervention on institutional delivery would be an over-estimate. The sampling strategy used for the study can be criticized for introducing bias because the interviewers may avoid hard-to-reach areas and non-responders may not be revisited [35]. Nonetheless, since the sampling bias was similar in the two study arms, and since the intervention effects were the differences in the outcomes of interest between intervention and comparison groups, the sampling bias was likely cancelled out from the intervention effect estimates.

## Conclusions

This study indicates initiatives targeted at improving maternal and neonatal health services appear to work better where community engagement is part of the quality improvement approach. The PCQI strategy is associated with increased service utilization of maternal care services, although their effect on postnatal and neonatal care is less clear. Any quality improvement approaches on maternal and newborn care practices should consider engaging communities to complement their strategies. Further studies could be conducted to assess the determinant factors for successful community engagement in improving quality of care including client confidence, trust and perception towards the health system. Moreover, cost-effectiveness of the PCQI strategy and the effects on postnatal and neonatal care, in addition to a wider set of outcomes, should be explored further.

## Additional file

Additional file 1: Standardized mean differentials of the co-variates between the two study arms, before and after matching. This table shows the standardized differences in the co-variates between intervention and comparison group respondents before and after matching for the seven PSM models. (DOCX 52 kb)

## Abbreviations

ANC: Antenatal care
ATE: Average treatment effect
BEmONC: Basic emergency obstetric and newborn care
CBDDM: Community-based data for decision-making
CI: Confidence interval
HEW: Health Extension Worker
L10 K: Last ten kilometers
PCQI: Participatory community quality improvement
PNC: Postnatal care
PSM: Propensity score matching
WDA: Women’s development army
