# Supplementary material for: Effects of a participatory community quality improvement strategy on improving household and provider health care behaviors and practices: a propensity score analysis
Source: BMC Pregnancy Childbirth. 2018 Sep 24;18(Suppl 1):364. doi: 10.1186/s12884-018-1977-9 (PMC6157250; doi:10.1186/s12884-018-1977-9)
Supplement: Supplementary file 1 — Standardized mean differentials of the co-variates between the two study arms, before and after matching. This table shows the standardized differences in the co-variates between intervention and comparison group respondents before and after matching for the seven PSM models. (DOCX 52 kb) [file 12884_2018_1977_MOESM1_ESM.docx]

**Additional file 1**

**Standardized mean differentials of the co-variates between the two study arms, before and after matching**

| Independent variables | ANC4+ | | Neonatal tetanus protection | | Complete ANC | | Institutional deliveries | | Early postnatal care | | Clean cord care | | Immediately initiating breastfeeding | |
| --- | --- | --- | --- | --- | --- | --- | --- | --- | --- | --- | --- | --- | --- | --- |
|  | Raw | Matched | Raw | Matched | Raw | Matched | Raw | Matched | Raw | Matched | Raw | Matched | Raw | Matched |
| Baseline estimates for the outcome | 0.294 | -0.020 | 0.652 | 0.007 | 0.344 | 0.031 | 0.697 | 0.043 | 0.212 | 0.012 | 0.170 | 0.092 | -0.109 | -0.035 |
| Women's age | -0.126 | -0.011 | -0.126 | -0.038 | -0.124 | 0.041 | -0.124 | -0.046 | -0.124 | -0.060 | -0.016 | -0.021 | -0.016 | -0.007 |
| Education | 0.254 | 0.007 | 0.254 | -0.051 | 0.256 | -0.075 | 0.256 | -0.026 | 0.256 | 0.060 | 0.251 | 0.018 | 0.251 | 0.099 |
| Marital status | -0.075 | -0.017 | -0.075 | -0.052 | -0.075 | 0.043 | -0.075 | -0.021 | -0.075 | -0.034 | -0.166 | 0.009 | -0.166 | -0.082 |
| Number of children | -0.199 | -0.064 | -0.199 | -0.010 | -0.195 | 0.035 | -0.195 | -0.022 | -0.195 | -0.078 | -0.086 | -0.035 | -0.086 | -0.076 |
| Religion |  |  |  |  |  |  |  |  |  |  |  |  |  |  |
| Protestant | -0.021 | -0.063 | -0.021 | -0.040 | -0.023 | 0.005 | 0.008 | 0.042 | -0.023 | -0.049 | -0.241 | -0.057 | -0.241 | 0.017 |
| Muslim | 0.010 | 0.032 | 0.029 | -0.066 | 0.034 | -0.037 | -0.023 | 0.061 | 0.015 | 0.028 | 0.062 | -0.027 | 0.062 | -0.094 |
| Other |  |  | -0.098 | 0.044 | -0.098 | 0.025 |  |  |  |  | -0.036 | 0.006 | -0.036 | 0.042 |
| Wealth quintile |  |  |  |  |  |  |  |  |  |  |  |  |  |  |
| Fourth | -0.044 | 0.074 | -0.044 | 0.057 | -0.038 | 0.071 | -0.038 | -0.019 | -0.038 | 0.020 | 0.064 | 0.014 | 0.064 | -0.096 |
| Middle | -0.291 | -0.038 | -0.291 | -0.079 | -0.292 | -0.043 | -0.292 | -0.036 | -0.292 | -0.004 | -0.187 | -0.053 | -0.187 | 0.018 |
| Second | 0.003 | 0.009 | 0.003 | 0.008 | 0.001 | -0.005 | 0.001 | 0.035 | 0.001 | 0.031 | 0.023 | 0.029 | 0.023 | 0.098 |
| Highest | 0.311 | -0.026 | 0.311 | 0.046 | 0.309 | -0.039 | 0.309 | 0.024 | 0.309 | -0.020 | 0.165 | 0.057 | 0.165 | -0.021 |
| Distance to health facility | -0.263 | -0.054 | -0.263 | -0.033 | -0.265 | -0.040 | -0.265 | 0.025 | -0.265 | -0.023 | -0.249 | 0.096 | -0.249 | 0.063 |
| Region |  |  |  |  |  |  |  |  |  |  |  |  |  |  |
| Amhara | 0.041 | -0.069 | 0.041 | 0.005 | 0.040 | 0.035 | 0.040 | -0.080 | 0.040 | -0.018 | 0.108 | -0.076 | 0.108 | -0.032 |
| Oromia | -0.098 | -0.003 | -0.098 | -0.034 | -0.100 | 0.005 | -0.100 | -0.005 | -0.100 | 0.032 | -0.038 | -0.074 | -0.038 | 0.011 |
| SNNP | 0.168 | -0.044 | 0.168 | -0.029 | 0.172 | -0.032 | 0.172 | -0.012 | 0.172 | -0.066 | 0.003 | 0.005 | 0.003 | -0.035 |
| Population to HEW ratio | 0.078 | -0.047 | 0.078 | -0.052 | 0.078 | -0.025 | 0.078 | 0.021 | 0.078 | -0.073 | -0.086 | 0.027 | -0.086 | 0.019 |
| ANC = Antenatal care; ANC 4+ = Received four or more antenatal care visits; HEW = Health extension worker; SNNP = Southern Nations, Nationalities and Peoples | | | | | | | | | | | | | | |
